# Effect of two-level decompressive procedures on the biomechanics of the lumbo-sacral spine: an *ex vivo* study

**DOI:** 10.3389/fbioe.2024.1400508

**Published:** 2024-07-09

**Authors:** Sara Montanari, Elena Serchi, Alfredo Conti, Giovanni Barbanti Bròdano, Rita Stagni, Luca Cristofolini

**Affiliations:** ^1^ Department of Industrial Engineering, Alma Mater Studiorum—Università di Bologna, Bologna, Italy; ^2^ Neurosurgery Unit, IRCCS Istituto delle Scienze Neurologiche di Bologna, Bologna, Italy; ^3^ Department of Biomedical and Neuromotor Sciences (DIBINEM), Alma Mater Studiorum—Università di Bologna, Bologna, Italy; ^4^ Spine Surgery Department, IRCCS Rizzoli Orthopaedic Institute, Bologna, Italy; ^5^ Department of Electrical, Electronic and Information Engineering “Guglielmo Marconi”, Alma Mater Studiorum—Università di Bologna, Bologna, Italy

**Keywords:** lumbo-sacral spine, biomechanics, decompressive surgeries, hemilaminectomy, laminectomy, range of motion, intervertebral disc, strain distribution

## Abstract

Hemilaminectomy and laminectomy are decompressive procedures commonly used in case of lumbar spinal stenosis, which involve the removal of the posterior elements of the spine. These procedures may compromise the stability of the spine segment and create critical strains in the intervertebral discs. Thus, this study aimed to investigate if decompressive procedures could alter the biomechanics of the lumbar spine. The focus was on the changes in the range of motion and strain distribution of the discs after two-level hemilaminectomy and laminectomy. Twelve L2-S1 cadaver specimens were prepared and mechanically tested in flexion, extension and both left and right lateral bending, in the intact condition, after a two-level hemilaminectomy on L4 and L5 vertebrae, and a full laminectomy. The range of motion (ROM) of the entire segment was assessed in all the conditions and loading configurations. In addition, Digital Image Correlation was used to measure the strain distribution on the surface of each specimen during the mechanical tests, focusing on the disc between the two decompressed vertebrae and in the two adjacent discs. Hemilaminectomy did not significantly affect the ROM, nor the strain on the discs. Laminectomy significantly increased the ROM in flexion, compared to the intact state. Laminectomy significantly increased the tensile strains on both L3-L4 and L4-L5 disc (*p* = 0.028 and *p* = 0.014) in ipsilateral bending, and the compressive strains on L4-L5 intervertebral disc, in both ipsilateral and contralateral bending (*p* = 0.014 and *p* = 0.0066), with respect to the intact condition. In conclusion, this study found out that hemilaminectomy did not significantly impact the biomechanics of the lumbar spine. Conversely, after the full laminectomy, flexion significantly increased the range of motion and lateral bending was the most critical configuration for largest principal strain.

## 1 Introduction

**FIGURE 1:**
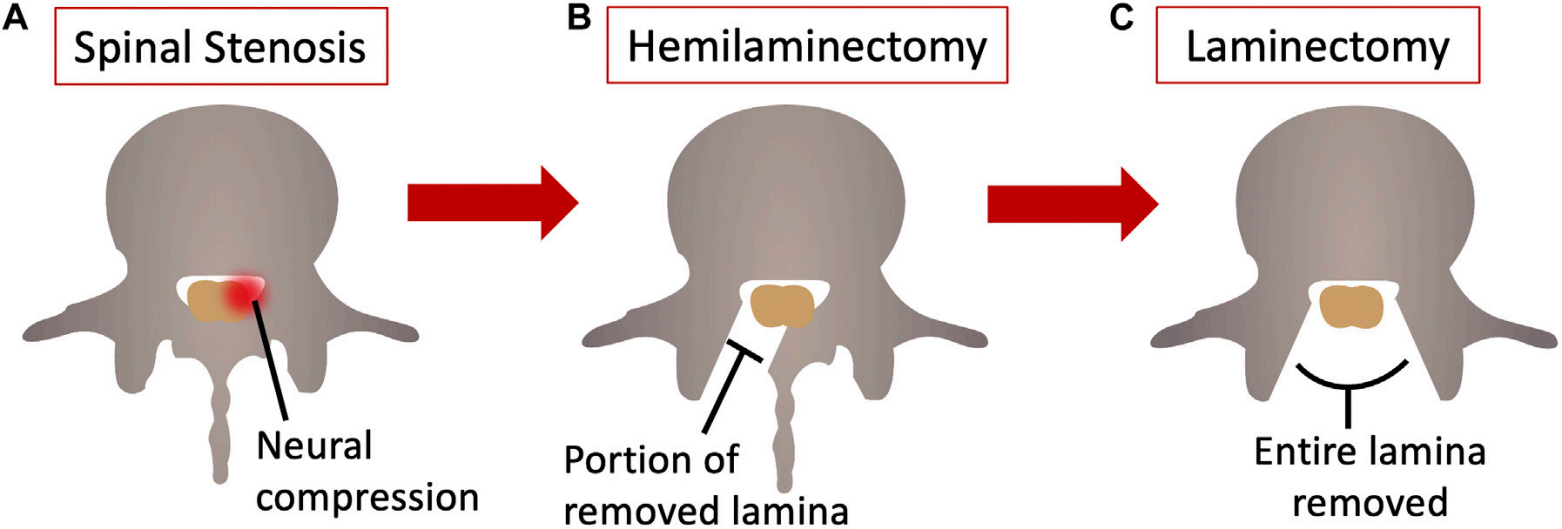
Axial view of a lumbar vertebra **(A)** with lumbar spinal stenosis; **(B)** after the hemilaminectomy, and **(C)** after the laminectomy. Figure published on Figshare repository (https://doi.org/10.6084/m9.figshare.25382323).

Lumbar spinal stenosis refers to the narrowing of the space within the spinal canal, nerve canals, or neural foramina, due to congenital, degenerative factors or a combination of both and resulting in compression of the neural elements of the lumbar spine (Arbit and Pannullo, 2001). This condition is prevalent among elderly individuals (Arbit and Pannullo, 2001; Issack et al., 2012; Abbas et al., 2013; Smith et al., 2014) and is, actually, the leading cause of lumbar spine surgery in adults over 65 years old (Deyo et al., 2005). Studies by Weinstein et al. (2008, 2010), demonstrated that surgical management are more effective in alleviating symptoms, pain, and improving function compared to simple conservative treatments, both in the short-term and up to 4 years post-surgery. Similar clinical outcomes were reported by (Atlas et al., 2000; Jarrett et a,. 2012; Atlas et al., 2000; Jarrett et al., 2012). Commonly used surgical procedures include decompression of the neural elements by hemilaminectomy and laminectomy, which involve the removal of part of the posterior elements of the spine combined with ligamentum flavum removal and different levels of lateral decompression of nerve roots (Estefan et al., 2024) (Figure 1). However, these procedures may compromise the stability of the affected spinal segment, potentially leading to complications such as spondylolisthesis, reported in 5.5% of laminectomy cases (Guha et al., 2015). Indeed, laminectomy could be expected to excessively sacrifice the posterior joints of the lumbar spine: to reduce the risk of instability, the surgeon must often face the dilemma of adding pedicular fixation to the decompressive surgical time. In these cases, a relatively simple procedure turns into a more complex one, often associated to surgical complications. Conversely, hemilaminectomy preserves the facet joints, the spinous process and both the supraspinous and interspinous ligaments. Therefore, hemilaminectomy is less likely to promote the onset of spondylolisthesis. To the authors’ best knowledge, no data are available stating the spondylolisthesis rate after the hemilaminectomy decompressive treatment.

The destabilizing effects of these procedures have been studied *ex vivo*, focusing on variation in the range of motion (ROM) of single functional spine units or longer spinal segments. (Fiss et al., 2021; Fiss et al., 2021) assessed the range of motion in the cervical spine after different decompressive treatments, including hemilaminectomy and laminectomy. They reported that C4 and C5 hemilaminectomy do not alter the stability of the cervical spine, and that laminectomy do not increase the range of motion in the tested loading configuration. Other studies in the literature focused on the lumbar spine. Delank et al., 2010, did not report an increase in the segmental mobility neither after hemilaminectomy, nor after laminectomy of L3 (Delank et al., 2010). Costa et al. (2018) found that bone-preserving laminectomy (with the preservation of facet joints and laminar bony bridge) has a limited biomechanical effect. They performed also a unilateral laminotomy, but not the hemilaminectomy. Additionally, they assessed the range of motion of the single motion segment, without assessing any effects on the adjacent segment (Costa et al., 2018). Lener et al. (2023), reported that the intersegmental motion of four-lumbar-vertebrae specimens significantly increased after laminectomy, as opposed to hemilaminectomy. The ROM of the adjacent motion segment was not significantly affected by the amount of decompression (Lener et al., 2023). Quint et al. (1998) simulated the laminectomy only on the L4 vertebra and measured the range of motion of the L4-L5 segment only (Quint et al., 1998). Lee et al. (2010), reported that the preservation of the central posterior osteoligamentous structures may provide a stabilizing effect in preventing post-decompression complications. They analysed both the total and intersegmental range of motion and stiffness in lumbar segment after a bilateral laminotomy and laminectomy at L2-L3, L3-L4 and L4-L5 (Lee et al., 2010). Finally, Smith et al., 2014, compared a minimally invasive hemilaminectomy of L4 (and of L5 partially) with the traditional laminectomy, removing in both cases the facet joints. Hamasaki et al. 2009, in 2009 conducted a cadaveric study specifically on lumbar functional spinal units, assessing only the stiffness of the motion segment. Fu et al. 2017, in 2017, evaluated strain distribution in the posterior articular processes using strain gauges following bilateral facetectomy and posterior fixation. Despite several biomechanical studies on decompressive surgical procedures, none have analysed the variation in strain distribution on the intervertebral disc, to the authors’ knowledge. Zander et al. 2003, in 2003, estimated stress and strain on the anulus fibrosus, as well as the range of motion, following simulated decompressive surgery at a single level using a finite element model. Investigations on the topic have been recently summarized in a review study (Ruspi et al., 2019).

While most studies have focused on single-level decompressive surgery, stenosis at multiple levels is more prevalent than strictly segmental stenosis, with L3-L4 and L4-L5 being the most commonly affected segments (Arbit and Pannullo, 2001). Moreover, alterations resulting from decompressive surgery can affect not only the anatomy at the operated level(s) but also the forces and stresses on adjacent levels, potentially leading to abnormal strains and damage to adjacent intervertebral discs. Therefore, past studies targeting only the single level where laminectomy was simulated only partially address concerns about the consequences of decompressive surgery.

Gawri et al., 2014; Alkhatib et al., 2014, reported that degenerative disc disease is associated with the release of inflammatory factors in intervertebral disc cells as a consequence of high strains (Alkhatib et al., 2014; Gawri et al., 2014). Although abnormal disc strains are one of the predictors of risk damage (Iatridis et al., 2005; Schmidt et al., 2009), alterations of the strain distribution in the intervertebral disc due to spine surgeries have seldom been quantified so far.

These decompressive treatments are suspected to alter the stability of the lumbar spine, and create critical strains in the discs directly involved, and in the ones adjacent to the treated levels. For this reason, it is worth to investigate if removal of different portion of the posterior spine could lead to significant loss of spine stability and/or to critical strains in the intervertebral discs. Therefore, the aim of this study was to investigate whether decompressive surgical procedures through hemilaminectomy and laminectomy could adversely affect the biomechanics of the lumbar spine, altering its mobility or creating critical strains in the intervertebral discs directly involved and in adjacent discs. Specifically, this study assessed changes in the range of motion of the lumbar spine and strain distribution of the intervertebral discs before and after two-level hemilaminectomy and laminectomy.

## 2 Materials and methods

For this study, twelve L2-S1 specimens were prepared for testing, leaving intact all the ligamentous structures. Two-level hemilaminectomy and laminectomy were sequentially performed on L4-L5 vertebrae by an expert surgeon. All the specimens were tested in the intact condition, after the hemilaminectomy and after the laminectomy under the same loading configurations, in flexion, extension, left and right lateral bending. The range of motion and the strain distribution were measured using Digital Volume Correlations (DIC) and compared among the three different conditions.

### 2.1 Ethics

This study was approved by the Bioethics Committee of the University of Bologna (Prot. n. 113043 of 10 May 2021), and was performed in line with the principles of the Declaration of Helsinki.

### 2.2 Specimens preparation

Twelve fresh frozen human L2-S1 spine specimens (7 males and 5 females, median age 74 years, median BMI 30 kg/m^2^) were harvested from twelve fresh cadavers (Table 1). All the spine cadavers were obtained from an ethically approved donation program (Anatomy Gift Registry, AGR). Spines were excluded if the donor was subjected to previous vertebral fractures, underwent a previous spine surgery or died with metastatic cancer. Additionally, the spine was excluded in case of severe deformity, or if tested positive for contagious diseases (HIV, hepatitis, syphilis, or COVID-19). All specimens were frozen al −28°C and sealed in a double plastic bag until prepared and tested.

**TABLE 1:** Details about the *ex vivo* specimens. The first columns summarize the donors’ information. The last column indicates if relevant osteophytes were present, and how they were treated. Median and interquartile range (IQR) are reported for age and BMI.

Specimen	Sex	Age (years)	BMI (kg/m^2^)	Cause of death	Presence of relevant osteophytes
#1	M	70	32	Myocardial infarction	L5-S1 left and right, removed
#2	M	79	25	Stroke	L4-L5 left, removed; bridge L5-S1 left, removed
#3	F	75	40	Arteriosclerotic cardiovascular disease	None
#4	F	82	23	Cardiac arrest	None
#5	M	76	22	Arteriosclerotic cardiovascular disease	None
#6	M	74	27	Anoxic brain injury	L4-L5 left and right, removed
#7	F	56	48	Septic shock	L5-S1 right, removed
#8	F	75	51	Sepsis	None
#9	M	62	17	Blunt force trauma	L2-L3 right, removed
#10	M	72	26	End stage liver disease	None
#11	F	62	43	Glioblastoma	L5-S1 left and right, removed
#12	M	73	36	Aspiration pneumonia	L5-S1 right, removed
Median	—	74	30	—	—
IQR	—	7	16	—	—

Computer tomography (CT) scans of the whole spines were taken (G.E. Revolution HD 1700, current: 80 mA, voltage: 120 kV, slice thickness: 0.625 mm) to assess the status of the spine in terms of disc degeneration, osteophytes, calcified ligaments, and bone fusions, and to confirm the absence of previous fractures, surgeries, tumors or metastases. After preparation of the specimens for biomechanical testing, further CT images of each specimen were acquired (with the same parameters, and including a densitometric calibration with a European Spine Phantom, ESP) to define in which cases osteophytes should be removed, and to measure the anatomical dimension of the L4 vertebra to compute the offset for load application.

The specimens were thawed in water at room temperature before preparation. Skin, fat and muscles were carefully removed, while the intervertebral discs, facet joint capsules and the ligamentous structures were left intact to preserve the natural kinematics (Widmer et al., 2020). The L2-S1 segment was extracted from each whole spine. To ensure that all the specimens were mounted reproducibly (Wilke et al., 1998) and that mechanical loading was applied properly to all the specimens, each spine segment was aligned with the L4 vertebra horizontal in both the sagittal and transverse plan, using a six-degree-of-freedom clamp and following a reproducible and suitable published procedure (Danesi et al., 2014).

Then, the upper half of the cranial vertebra (L2) and the lower half of the caudal vertebra (sacrum) were embedded in acrylic resin (Technovit 4071, Heraeus Kulzer, Wehrheim, Germany) to mount the specimens in the loading device. Based on the CT scans, osteophytes were assessed by a surgeon and removed in case of bridging or obstructing the kinematic motion (Table 1).

### 2.3 Surgical procedures

All the twelve specimens underwent stepwise surgical decompression starting from the intact condition. All the surgical procedures were performed by an expert surgeon of one hospital partner of this study. The surgeon simulated the exact surgery which is carried out on patients suffering from lumbar spinal stenosis.

#### 2.3.1 Hemilaminectomy

**FIGURE 2:**
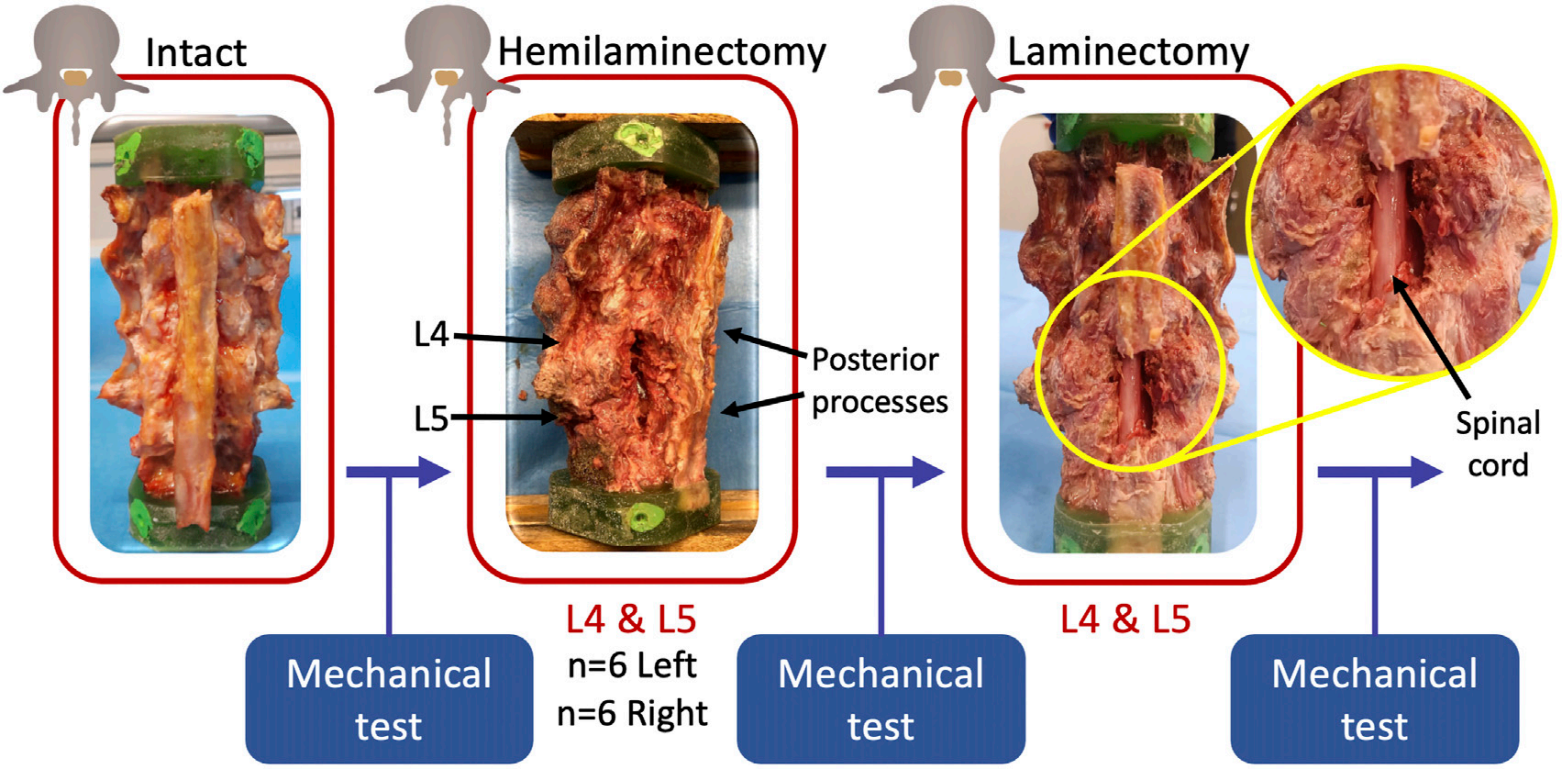
Workflow of the study. After the preparation of the intact specimens, a two-level hemilaminectomy was performed by an expert surgeon randomly on the left or on the right side. Finally, a laminectomy on the same two levels was performed. Each specimen was mechanically tested under the same loading configurations in each condition. Figure published on Figshare repository (https://doi.org/10.6084/m9.figshare.25323784).

After being tested in the intact condition in all the loading configurations, a two-level hemilaminectomy was performed on all the specimens (Figure 2). An expert surgeon simulated the two-level hemilaminectomy on the L4 and L5 vertebrae. After the identification of the L4 and L5 vertebrae on the posterior side, the L4 lamina was removed starting to the medial border (the junction between the lamina and the posterior process) by means of Kerrison rongeurs. In this way, the ligamentum flavum and the epidural fat were exposed in order to free the canal without damaging the dural sac. The decompression was extended laterally, until the junction with the facet joint, without damaging the joint capsule. The same procedure was reproduced on the L5 vertebra. The two-level hemilaminectomy was randomly performed on the left side on six specimens, and on the right side on the remaining six (Table 2).

**TABLE 2:** The central columns show the offset applied in flexion, extension, and lateral bending. Three specimens (indicated with *) had nearly no lordosis and it was necessary to apply an offset of 150% of the antero-posterior length of the L4 vertebra in extension instead of 100%. The last column reports the side of the spine where the hemilaminectomy was randomly performed (6 left, 6 right). Median and interquartile range (IQR) are reported for the offsets.

Specimen	Offset			Side of hemilaminectomy
	Flexion (mm)	Extension (mm)	Lateral bending (mm)	
#1	10.2	34.0	21.9	Left
#2	10.6	35.5	24.7	Right
#3	9.2	45.8*	21.8	Right
#4	8.7	43.5*	18.7	Left
#5	8.6	42.8*	22.3	Left
#6	12.6	42.0	25.6	Right
#7	8.8	29.2	17.9	Right
#8	8.9	31.6	19.3	Right
#9	10.4	34.7	23.1	Right
#10	9.4	31.4	21.3	Left
#11	9.4	31.3	21.3	Left
#12	10.5	35.0	21.9	Left
Median	9.4	34.0	21.9	—
IQR	1.6	3.6	1.7	—

#### 2.3.2 Laminectomy

After being tested in the hemilaminectomy condition in all the loading configurations, a full laminectomy was performed on all the specimens by the same surgeon (Figure 2). First, the supraspinous and interspinous ligaments between L3 and L4 and between L5 and sacrum were cut by means of a scalpel in order to remove the spinous process of the L4 and L5 vertebrae. The remaining bony structures were then removed in order to expose the ligamentum flavum and the epidural fat, and a hemilaminectomy, as described above, was performed on the L4-L5 laminae not previously removed.

### 2.4 Mechanical tests

**FIGURE 3:**
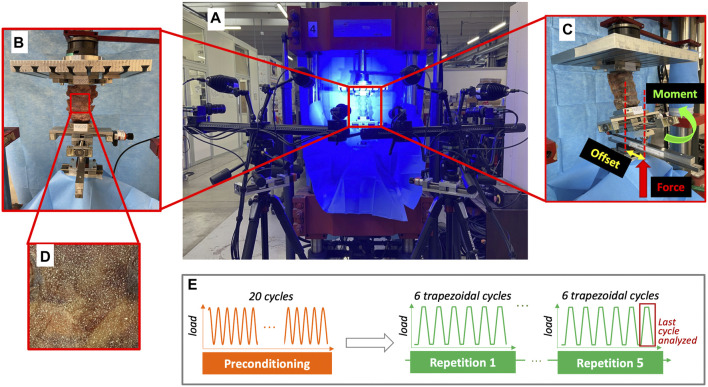
**(A)** Overview of the experimental setup with the four cameras of the Digital Image Correlation system framing the specimen mounted in the testing machine. **(B)** Frontal zoomed image showing how the specimen was mounted on the testing machine. Below the specimen, the micrometric adjustable bidirectional slide is visible, which was used to impose the desired offset of the force, which was delivered through a spherical joint, mounted on top of the low-friction rail. **(C)** The lateral view shows the application of the force with an offset by means the micrometric adjustable bidirectional slide, in order to deliver the target moment. **(D)** A zoomed detail on the specimen surface shows the white random speckle pattern sprayed on all the surface of the vertebrae and of the intervertebral discs. **(E)** The experimental testing sequence, which was replicated in all the different loading configurations, included a pre-conditioning and five repetitions of 6-cycle test. The red square highlights the last cycle of each repetition, where the data were extracted and analyzed. Figure published on Figshare repository (https://doi.org/10.6084/m9.figshare.25315168).

Each specimen was mechanically tested in flexion, extension and both left and right lateral bending, under the same testing conditions, in the intact condition, and after the simulation of the two-level hemilaminectomy and laminectomy. Each test was performed in displacement control by means of a uniaxial servo-hydraulic testing machine (Instron 8500 controller, Instron, United Kingdom) equipped with a 10 kN load cell. During each mechanical test, a combination of force and bending was applied, so as to reach the target moment of 2.5 Nm, with an initial preload of 20 N. A relatively low bending moment was intentionally chosen, to avoid the risk of damage during repeated testing before and after surgery. The cranial extremity of the specimen (L2 vertebra), embedded in the acrylic pot, was rigidly attached to the actuator of the testing machine by means of a metallic plate (Figure 3). In order not to constrain the relative motion of the specimen and enable the specimen to follow its natural motion, the caudal extremity of the specimen was linked to a spherical joint moving along a low-friction rail. In this way, free rotations and translations in the horizontal plane were allowed. A micrometric adjustable bidirectional slide allowed to apply the force with the desired offset with respect to the center of the L4 vertebra, in order to reach the target moment. An anterior, posterior or lateral offset were imposed to generate flexion, extension or lateral bending respectively. The offset was computed on the specific anatomy of each specimen, from the CT images, as a percentage of the length and width of the L4 vertebra (Table 2). In particular, an offset of 30% of the antero-posterior length of the vertebra was applied in case of flexion, and an offset of 100% of the antero-posterior length was decided in extension, as the lumbar spine is more flexible in flexion compared to extension. However, in three specimens the lordotic curvature was nearly absent, and the offset was increased to 150% of the antero-posterior length of L4. Lastly, an offset of the 50% of the right-left width of the L4 vertebra was applied for both the left and right lateral bending.

To generate a combination of force and bending, for each loading configuration, the force was applied to the specimen with the desired offset by adjusting the micrometric bidirectional slide (in the anterior direction for flexion, posterior direction for extension, and left and right direction for left and right direction, respectively) with respect to the center of the L4 vertebra. The force was applied by the actuator to the caudal extremity of the specimen. The spherical joint between the micrometric slide and the actuator allowed each specimen to bend in the direction defined by the application of the offset. Before starting each test, we loaded the specimen until the applied force combined with the actual total offset (the sum of the initial offset imposed by the micrometric slide, and of the displacement of the center of the L4 vertebra under load with respect to the unloaded condition) reached the target moment of 2.5 Nm. This force value was then applied during the repetitions of the actual test.

A preconditioning consisting of 20 sinusoidal cycles at 0.5 Hz was performed before the test of each different loading configuration to minimize the effects of viscoelasticity (Techens et al., 2020; Techens et al., 2022). Subsequently, the actual test consisted of 6 trapezoidal cycles (Figure 3), where the loading ramp lasted 1.0 s, the maximum load was held constant for 0.3 s (to allow acquisition of stable images, see below), and then the specimen was unloaded in 0.5 s (Techens et al., 2022). Each test was repeated 5 times on each specimen to assess the repeatability. In particular, the coefficient of variation was computed for each parameter as the ratio between the standard deviation and the average. The data from the last three repetitions were used for analysis and averaged. In each repetition, the first three cycles were sufficient for minimizing the viscoelastic response (Wilke et al., 1998; Cottrell et al., 2006), the subsequent cycles being nearly identical in terms of loads and displacements (Techens et al., 2022). So, the data from the last cycle of each repetition were extracted and analyzed.

All the tests were performed at room temperature, and specimens were wrapped in wet paper to keep hydration of tissues while the test rig was adjusted for the different loading configurations (Wilke et al., 1998).

### 2.5 Data acquisition with the digital image correlation

During each test, the displacement and strain distribution (Palanca et al., 2018) on the surface of the specimen (including both the vertebrae and the intervertebral discs) were measured using a state-of-the-art Digital Image Correlation (DIC). The DIC system (Aramis Adjustable 12M, GOM, Braunschweig, Germany) included four high-resolution cameras with a resolution of 12Mpixel (4096 × 3000) and four metrology-quality lenses Titanar B 75 (f 4.5), and a light system with 4 LEDs light with 10° light cone (Figure 3). Thus, both the anterior and lateral sides of each specimen were acquired simultaneously throughout the tests. Before each test, the system was calibrated with a calibration target (Type CP40/200/101296t GOM, Aramis, Braunschweig, Germany). Images during the relevant loading cycles were acquired at 25 frames per second.

The DIC system allows to measure the strain distribution thanks to a random speckle pattern on the surface of the specimen. For this reason, a white pattern was sprayed on the antero-lateral surface of each specimen (Figure 3) using a water-based acrylic paint (Q250201 Bianco Opaco, Chreon, Italy). The air pressure, airflow, dilution, and distance were optimized to achieve the desired size of the speckle dots, following a published procedures (Lionello and Cristofolini, 2014; Lionello et al., 2014; Palanca et al., 2015). In particular, the following parameters were set: airbrush pressure of 1 bar, airflow with 3 turns of the screw in the airbrush, 20 mL of white paint were diluted with 8 mL of water, the paint was sprayed at the distance of 500 mm from each specimen. The speckle pattern was prepared at room temperature. A zero-strain analysis was performed to assess the intrinsic uncertainties of the DIC measurements on consecutive unloaded images. Five images of the unloaded specimen were acquired to assess the uncertainties of the measurements (Palanca et al., 2018). If no loads are applied, no displacements and strain should be theoretically observed. So, any value of displacement and strain different from zero should be accounted as measurement error, and were quantified to estimate the reliability of the DIC measurements under loads. A facet size of 34 pixels, a grid spacing of 19 pixels with a spatial medial filter of the 5th order and a temporal average filter of the 2nd order was chosen as the optimal parameters after testing different combinations of facet size, grid spacing and filtering. This corresponded to a spatial resolution of ≈6 mm.

### 2.6 Data analysis

Data were extracted and analyzed at the stage where the target moment of 2.5 Nm was reached, in the last loading ramp. Data from the last three repetitions were averaged for each condition and each loading configuration. Changes in the mobility of the lumbar spine were evaluated by means of the comparison of the range of motion (ROM) of the entire L2-sacrum segment. ROM was measured as the relative rotation of sacrum with respect to the L2 vertebra in the sagittal plane in case of flexion and extension, and in the coronal plane for lateral bending. This measurement was performed using the DIC dedicated software (Aramis Professional 2019), which calculated the relative rotation of two “point components” identified on the respective vertebrae. To quantify the alterations on the strain distribution in the intervertebral discs, the maximum (ε1, tensile) and minimum (ε2, compressive) principal strain field were measured on the entire anterior and lateral surface of each disc by means of the DIC dedicated software. To analyze if the two-level hemilaminectomy and laminectomy could have a worsening effect on the intervertebral discs, the analysis of the largest values of both the tensile and compressive strain was performed on the L4-L5 intervertebral disc. The same analysis was performed also on the intervertebral discs cranial (L3-L4) and caudal (L5-S1) the operated levels. The “largest” strain values were computed as the 95th and 5th percentile, in case of tensile and compressive strain respectively, to avoid local measurement artifacts.

Due to the inter-specimen variability, all the hemilaminectomy and laminectomy data were normalized with respect to the intact condition of each specimen.

### 2.7 Statistical analysis

A statistical analysis was performed in order to assess the significance of the difference between the two different decompressive procedures and the intact condition. The distribution of each data was tested for normality using the Shapiro-Wilk test (Supplementary Table S1). All the parameters are reported as median.

The effect of the spine condition (intact vs. hemilaminectomy vs. full laminectomy) was assessed using the Repeated Measures One-Way ANOVA with the Geisser-Greenhouse correction and the *post hoc* Tukey multiple comparison test, in case of normality. Otherwise, the Friedman test and the *post hoc* Dunn’s multiple comparison test was performed. This analysis was performed separately for each loading configuration, on the range of motion, largest tensile strain and largest compressive strain.

A *p-*value smaller than 0.05 was considered significant. All statistical analyses were performed using GraphPad Prism (Windows version 9.3.1, GraphPad Software, La Jolla, CA, United States).

## 3 Results

Image correlations and subsequent measurements were successfully performed for all the conditions and loading configurations. Due to a data loss, in the first six specimens the largest tensile and compressive strain on two of the intervertebral discs (L3-L4 and L5-S1) could not be retrieved for the intact and hemilaminectomy conditions. Despite that, in the worst case, at least six specimens were included in each analysis, allowing to achieve sufficient statistical power (Wilke et al., 1998).

Instead of analyzing only the last repetition, all the data were averaged among the last three repetitions for each condition and loading configuration, in order to increase the strength of each data (Techens et al., 2022). All data were then normalized with respect to the intact condition. In order to observe if the side toward the hemilaminectomy was performed impacted both the range of motion and the strain distribution on the lumbar spine in the lateral bending, results will be referred as *ipsilateral bending* (IpsiLB) if bending was towards the same side of the hemilaminectomy, or as *contralateral bending* (ContraLB) if bending was towards the opposite side of the hemilaminectomy.

### 3.1 Errors and repeatability analysis

**FIGURE 4:**
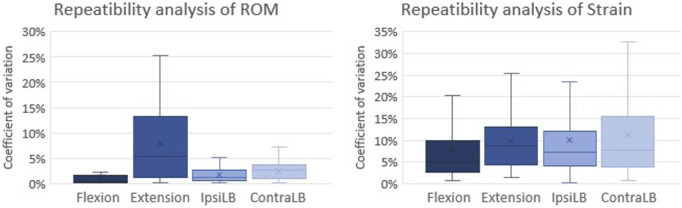
Analysis of the test repeatability: Coefficient of variation between repetitions on the same specimen for the Range of Motion (ROM) (left) and the strain (right). The coefficient of variation is reported as the average among all specimens for each loading configuration. The bottom of the box represents the first quartile (25th percentile) of the data; the horizontal line inside the box represents the median of the data and the top of the box represents the third quartile (75th percentile). The cross represents the mean of the data. The top and bottom whiskers include the maximum and the minimum data.

The zero-strain analysis indicated that the systematic error (accuracy) on the DIC-measured strain was less than 10 × 10^−6^, and the random error (precision) was less than 200 × 10^−6^. The intra-specimen repeatability tests showed that for the range of motion the coefficient of variation between repetitions on the same specimen was 1.3% (average among all specimens) in flexion, 7.9% in extension, 1.7% in ipsilateral bending and 2.5% in contralateral bending (Figure 4). For the strains, the intra-specimen repeatability analysis focused on largest measured strain value on the specimen surface: the coefficient of variation between repetitions was 7.8% (average of all specimens) in flexion, 10% in extension and ipsilateral bending and 11.3% in contralateral bending (Figure 4).

### 3.2 Range of motion

**FIGURE 5:**
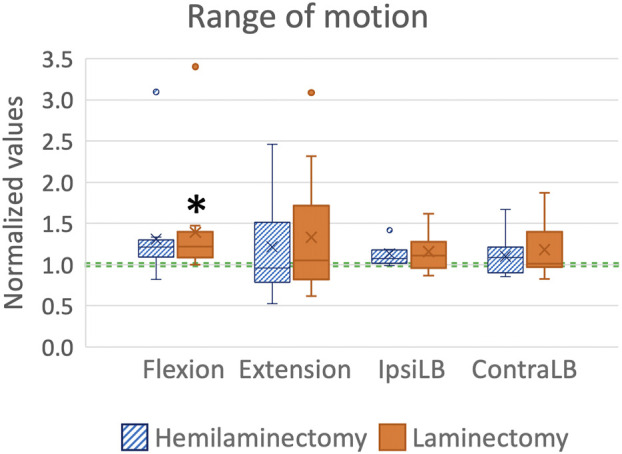
Range of motion (ROM) in flexion, extension, ipsilateral bending (IpsiLB) and contralateral bending (ContraLB) after the two-level hemilaminectomy and laminectomy. All the data are normalized with respect to the intact condition (green dashed line, corresponding to 1.0). A value larger than 1.0 indicates an increase of the range of motion with respect to the intact condition. The bottom of the box represents the first quartile (25th percentile) of the data; the horizontal line inside the box represents the median of the data and the top of the box represents the third quartile (75th percentile). The cross represents the mean of the data. The top and bottom whiskers include the maximum and the minimum data, excluding the outliers which are shown as dots. Statistically significant differences with respect to the intact condition (*p* < 0.05) are marked with an asterisk *.

A slightly increasing trend from the intact to the laminectomy condition was visible for the range of motion, but differences were significant only in some cases (Figure 5). Hemilaminectomy did not significantly alter the range of motion of the lumbar spine with respect to the intact condition (flexion: *p* = 0.106, ipsilateral bending: *p* = 0.073, contralateral bending *p* = 0.643, *post hoc* Tukey multiple comparison test, extension: *p* > 0.999, *post hoc* Dunn’s multiple comparison test). No statistically significant increases in ROM were observed between the hemilaminectomy and laminectomy (flexion: *p* = 0.187, ipsilateral bending: *p* = 0.470, contralateral bending: *p* = 0.277, *post hoc* Tukey multiple comparison test, extension: *p* > 0.999, *post hoc* Dunn’s multiple comparison test). Laminectomy significantly increased the range of motion with respect to intact condition by 22% in flexion (*p* = 0.016 One-Way ANOVA test and *p* = 0.028 *post hoc* Tukey’s multiple comparison test). Also, in ipsilateral bending the increasing ROM was statistically significant (*p* = 0.022 One-Way ANOVA test), but the *post hoc* test did not reveal any difference among the three conditions (*p* > 0.05 for all comparisons, *post hoc* Tukey’s multiple comparison test).

### 3.3 Qualitative analysis of the strain distribution on the intervertebral discs

**FIGURE 6:**
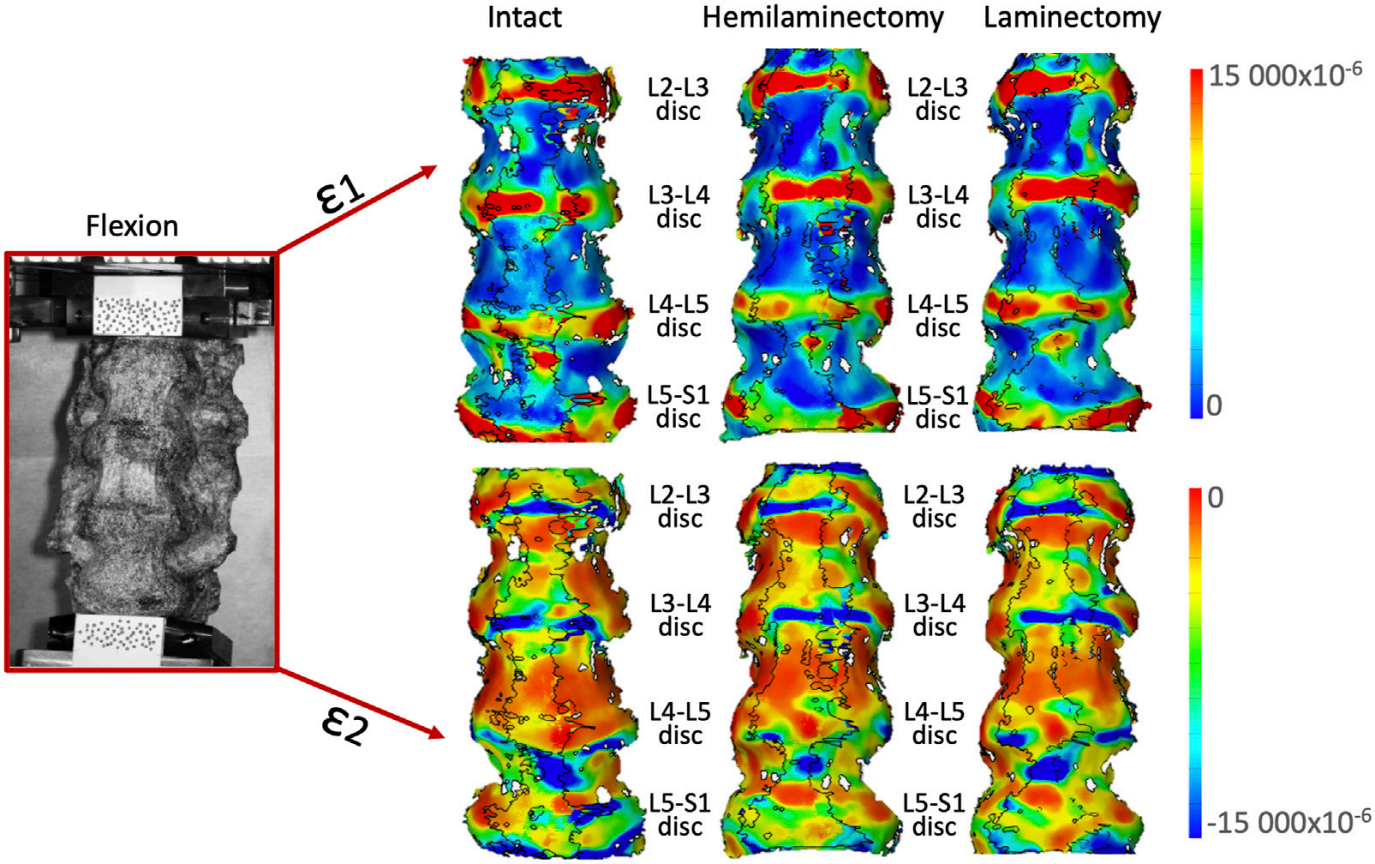
Flexion: Distributions of the maximum tensile (ε1, top) and minimum, compressive (ε2, bottom) strains on the anterior and lateral surface of a typical specimen. The intact condition is shown on the left, the hemilaminectomy in the middle and the laminectomy on the right. The image inside the red square on the left shows the specimens as viewed by the DIC cameras. The subsequent analyses focused on the intervertebral discs at the operated levels (L4-L5), and at the adjacent ones (mainly L3-L4 and L5-S1). Some strain peaks are also visible on the anterior longitudinal ligament in front of some of the vertebrae, but these were not considered as relevant for this study. Figure published on Figshare repository (https://doi.org/10.6084/m9.figshare.25315270).

In flexion (Figure 6) the highest tensile strains (ε1) were localized at mid-height of all the intervertebral discs, and showed a tendency to increase from the intact condition to the laminectomy. The compressive strains (ε2) were mainly located on the inferior endplate of the L2-L3, L3-L4 and L4-L5 intervertebral discs, and on both the endplates of the L5-S1 disc.

**FIGURE 7:**
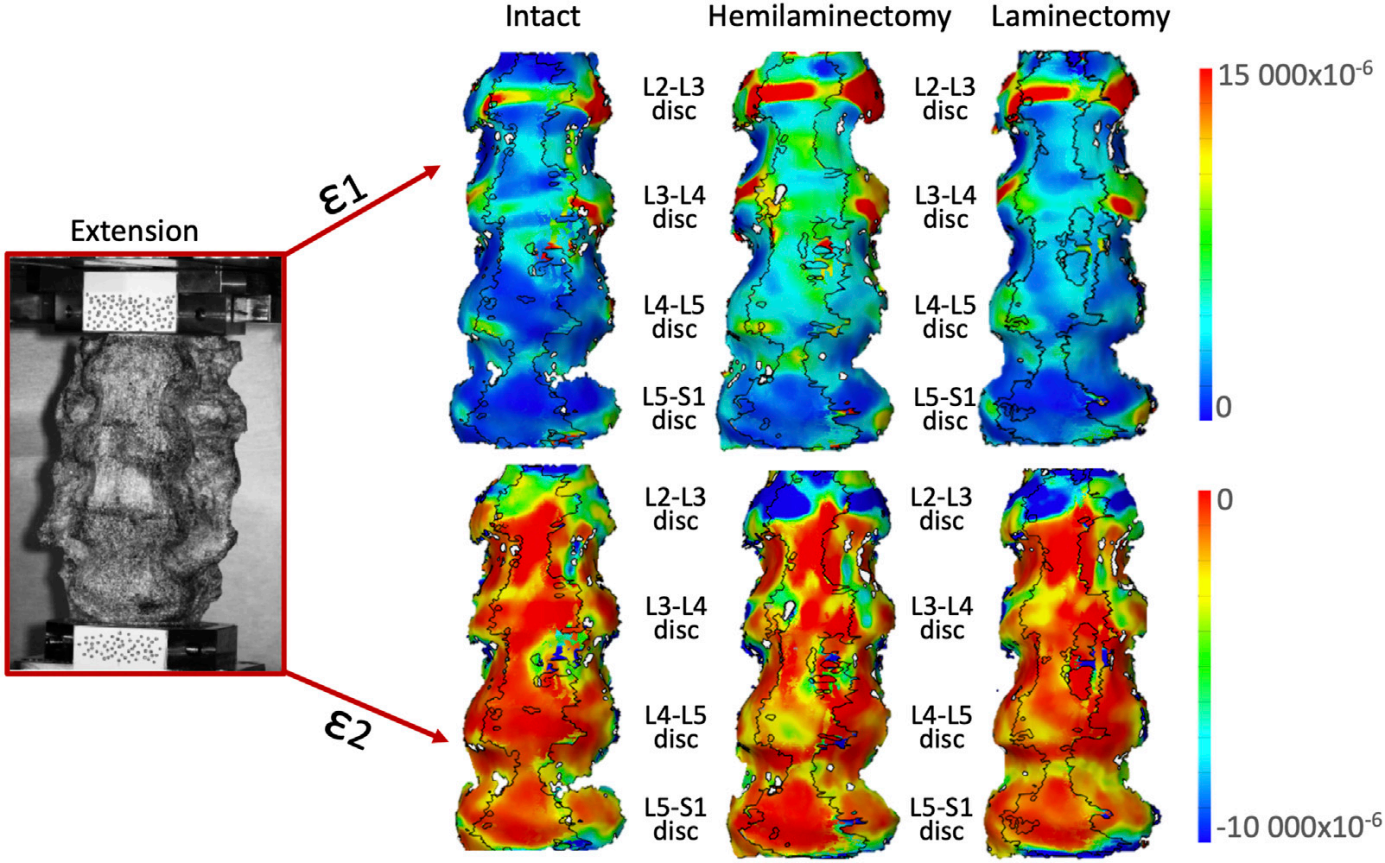
Extension: Distributions of the maximum tensile (ε1, top) and minimum, compressive (ε2, bottom) strains on the anterior and lateral surface of a typical specimen. The intact condition is shown on the left, the hemilaminectomy in the middle and the laminectomy on the right. The image inside the red square on the left shows the specimens as viewed by the DIC cameras. The subsequent analyses focused on the intervertebral discs at the operated levels (L4-L5), and at the adjacent ones (mainly L3-L4 and L5-S1). Some strain peaks are also visible on the anterior longitudinal ligament in front of some of the vertebrae, but these were not considered as relevant for this study. Figure published on Figshare repository (https://doi.org/10.6084/m9.figshare.25315402).

In extension (Figure 7), high tensile strains (ε1) were mainly located on the L2-L3 and L3-L4 intervertebral discs and. High tensile strains were visible also on the L4-L5 disc after both the hemilaminectomy and the laminectomy. Highest compressive strain (ε2) was visible especially on the L2-L3 intervertebral disc, and along the anterior part of the endplates. No remarkable variations in the distribution among the different conditions were observed.

**FIGURE 8:**
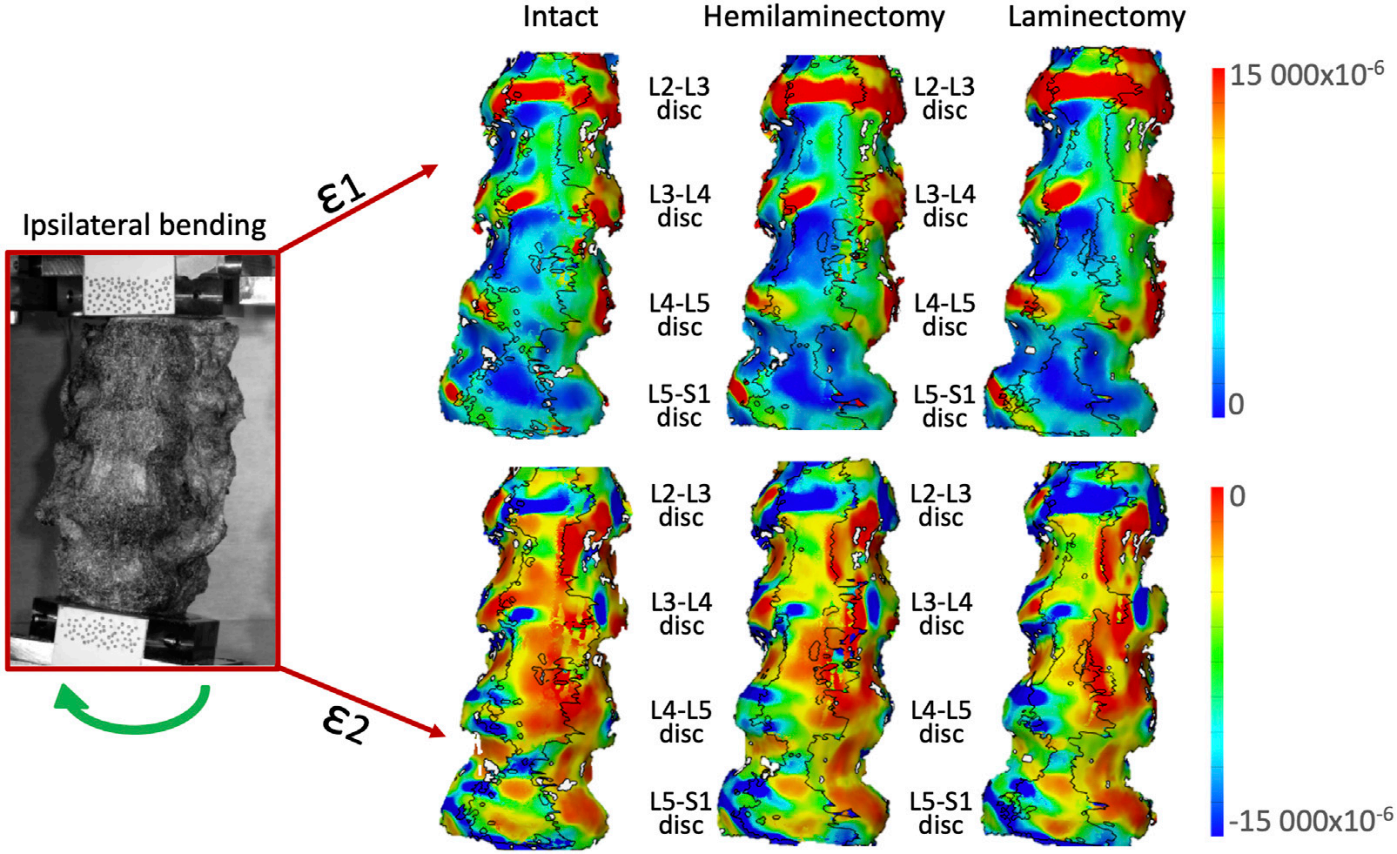
Ipsilateral bending: Distributions of the maximum tensile (ε1, top) and minimum, compressive (ε2, bottom) strains on the anterior and lateral surface of a typical specimen. The intact condition is shown on the left, the hemilaminectomy in the middle and the laminectomy on the right. The image inside the red square on the left shows the specimens as viewed by the DIC cameras. The subsequent analyses focused on the intervertebral discs at the operated levels (L4-L5), and at the adjacent ones (mainly L3-L4 and L5-S1). Some strain peaks are also visible on the anterior longitudinal ligament in front of some of the vertebrae, but these were not considered as relevant for this study. Figure published on Figshare repository (https://doi.org/10.6084/m9.figshare.25315513).

**FIGURE 9:**
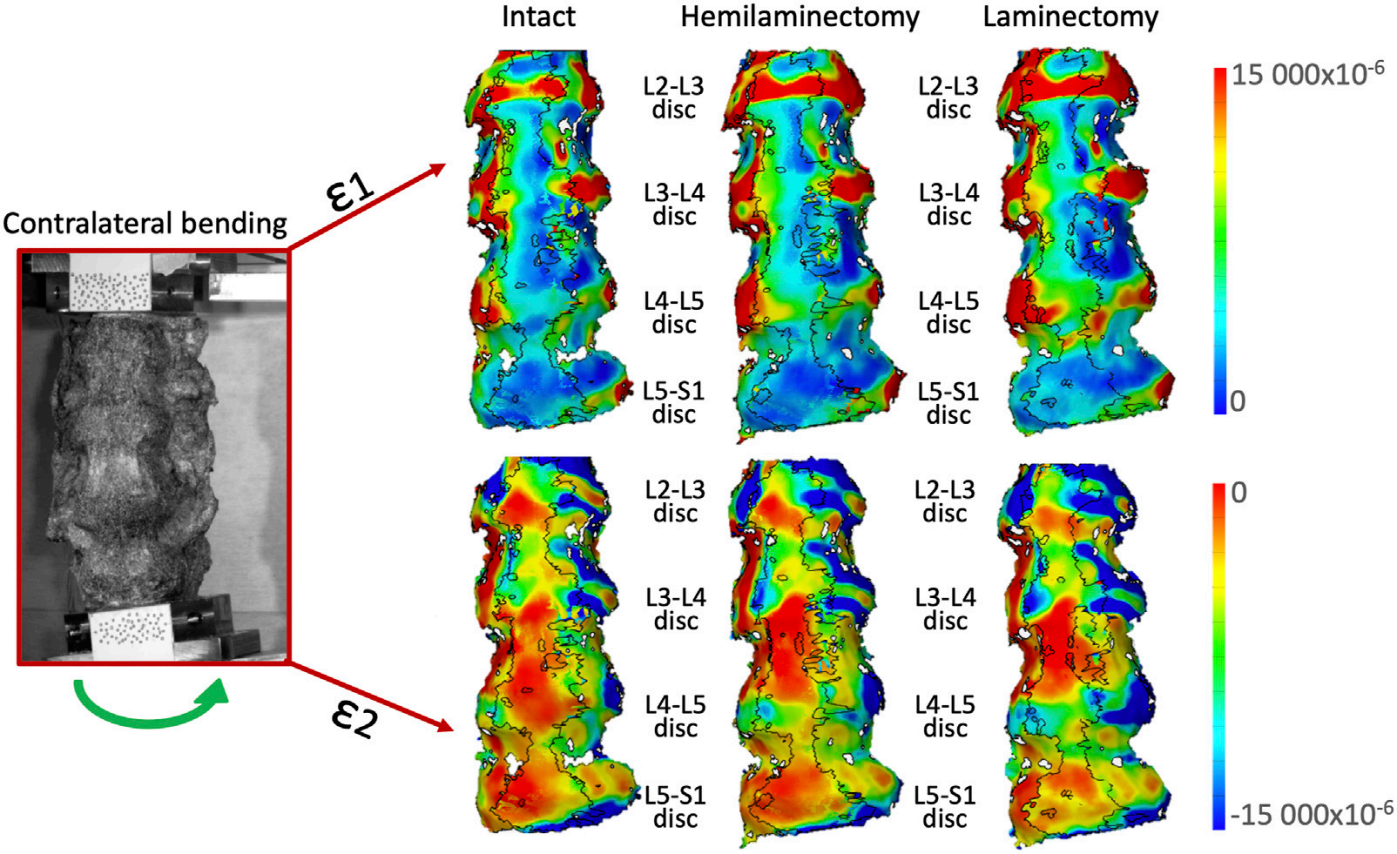
Contralateral bending: Distributions of the maximum tensile (ε1, top) and minimum, compressive (ε2, bottom) strains on the anterior and lateral surface of a typical specimen. The intact condition is shown on the left, the hemilaminectomy in the middle and the laminectomy on the right. The image inside the red square on the left shows the specimens as viewed by the DIC cameras. The subsequent analyses focused on the intervertebral discs at the operated levels (L4-L5), and at the adjacent ones (mainly L3-L4 and L5-S1). Some strain peaks are also visible on the anterior longitudinal ligament in front of some of the vertebrae, but these were not considered as relevant for this study. Figure published on Figshare repository (https://doi.org/10.6084/m9.figshare.25315564).

For lateral bending in both directions (Figures 8, 9), the highest high tensile strains (ε1) were located at mid-height of all the intervertebral discs, in the lateral part, towards the side where the bending was performed. High tensile strains were visible also in the lateral part on the opposite side of the bending including both the discs and the endplates. The areas affected by the highest strains seemed to enlarge from the intact to the laminectomy condition. Compressive strains (ε2) were located on the endplate along all the intervertebral discs. The highest compressive strains (ε1) were mainly visible on the lateral part of the endplates in the side of bending. The area affected by the highest strain increased after the laminectomy.

### 3.4 Quantitative analysis of the largest strains on the intervertebral discs

**FIGURE 10:**
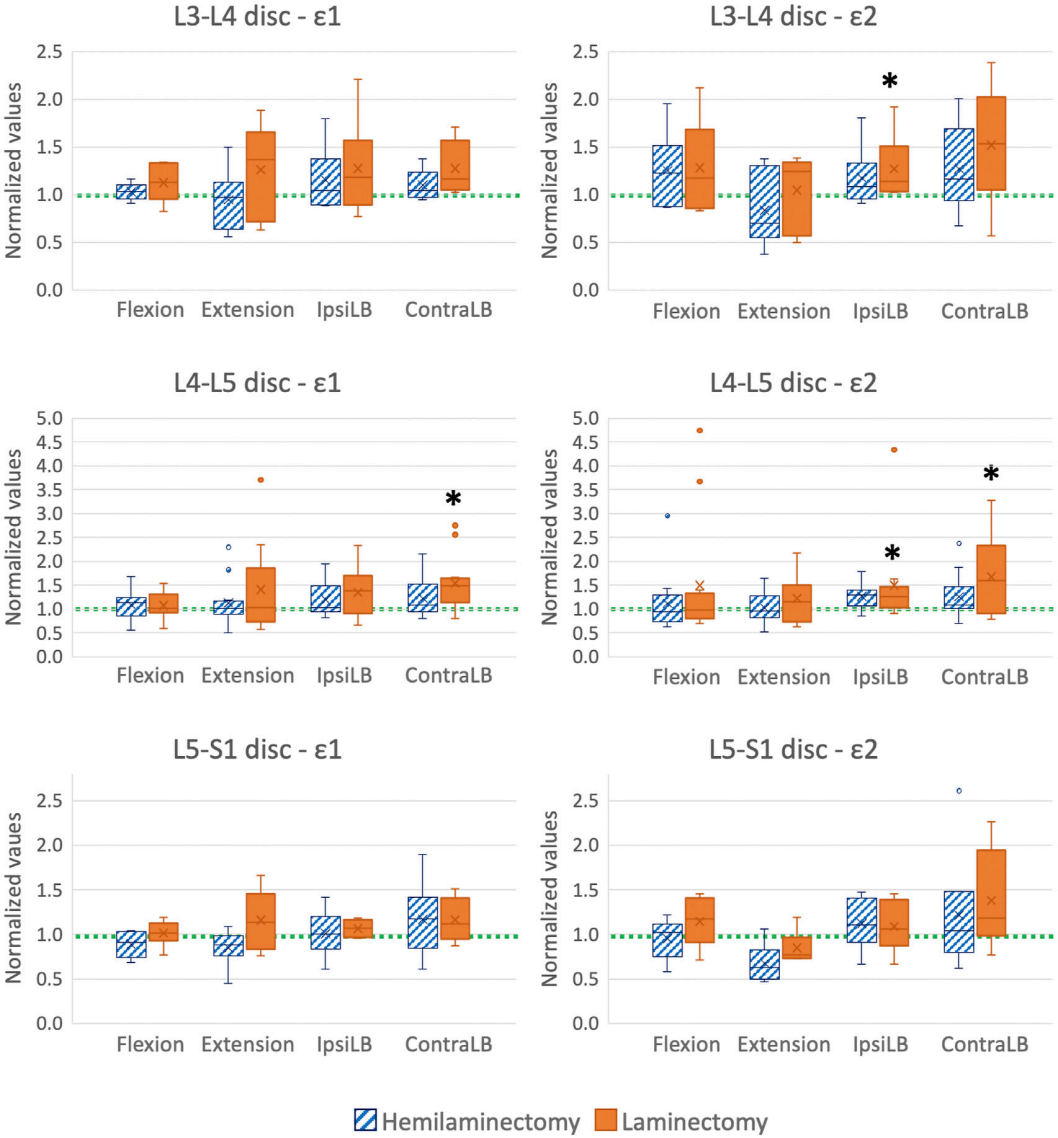
Largest tensile (ε1, left) and compressive (ε2, right) strains on the L3-L4 (top), L4-L5 (middle), and L5-S1 (bottom) intervertebral discs, in flexion, extension, ipsilateral bending (IpsiLB) and contralateral bending (ContraLB) after the two-level hemilaminectomy, and after laminectomy. L4-L5 intervertebral disc is the disc between the two decompressed vertebrae; L3-L4 and L5-S1 intervertebral discs are cranial and caudal to the decompressed levels. All the data are normalized with respect to the intact condition (dashed green line). A value larger than 1.0 indicates an increase of strain magnitude with respect to the intact condition. The bottom of the box represents the first quartile (25th percentile) of the data; the horizontal line inside the box represents the median of the data and the top of the box represents the third quartile (75th percentile). The cross represents the mean of the data. The top and bottom whiskers include the maximum and the minimum data, excluding the outliers which are shown as dots. Statistically significant differences with respect to the intact condition (*p* < 0.05) are marked with an asterisk *.

Focusing on the L4-L5 disc (between the two laminectomy levels), the laminectomy significantly increased both the largest tensile (48%: *p* = 0.0094 One-Way ANOVA test and *p* = 0.013 *post hoc* Tukey’s multiple comparison test) and compressive strain (74%: *p* = 0.0087 Friedman test and *p* = 0.0066 *post hoc* Dunn’s multiple comparison test) in contralateral bending with respect to the intact condition (Figure 10). Compressive strain on the L4-L5 disc significantly increased by 31% after laminectomy with respect to the intact condition in ipsilateral bending (*p* = 0.013 Friedman test and *p* = 0.013 *post hoc* Dunn’s multiple comparison test). No other statistically significant differences were observed on the strains on the L4-L5 disc.

Focusing on the disc cranial to the decompressive surgery (L3-L4), laminectomy significantly increased the compressive strain by 14% with respect to the intact condition in ipsilateral bending (*p* = 0.029 Friedman test and *p* = 0.028 *post hoc* Dunn’s multiple comparison test, Figure 10). Also, in contralateral bending, the compressive strain was significantly affected by the decompressive surgery (*p* = 0.029 One-Way ANOVA test), but the *post hoc* did not reveal any difference among the three conditions (*p* > 0.05 Tukey’s multiple comparison test). No significant differences were observed for the tensile strain on L3-L4 intervertebral disc.

Focusing on the disc caudal to the decompressive surgery (L5-S1), laminectomy did not significantly alter the tensile and compressive strains (Figure 10).

## 4 Discussion

This *ex vivo* study aimed to assess if and to what extent hemilaminectomy and laminectomy affect the biomechanics of the spine segment involved, including the adjacent levels. In particular, the focus was on the range of motion (which can be an indicator of instability) and on the strains in the intervertebral discs (which can be assumed as predictors of the risk of damage). For this purpose, twelve L2-S1 human specimens were tested in flexion, extension, and lateral bending after the simulation of a two-level hemilaminectomy and laminectomy to measure the alterations of the biomechanics of the lumbar spine with respect to the intact condition, by means of the Digital Image Correlation.

In this study, L2-S1 specimens were used, which are different from specimens of several previous studies reported in the literature, where we can find, for example, L1-L5 specimens (Delank et al., 2010; Lee et al., 2010), L1-Sacrum specimens (Smith et al., 2014), L2-L5 or L3-S1 specimens (Costa et al., 2018; Lener et al., 2023), L2-S2 specimens (Quint et al., 1998), or other specimens lengths. In each study the number of the vertebrae included in the prepared specimens is dependent to the aim and the analysis of the study. In the present study, the authors wanted to replicate as much as possible the surgical technique performed in the actual clinical practice, therefore hemilaminectomy and laminectomy were performed at the same L4 and L5 levels (Arbit and Pannullo, 2001). In addition, this study aimed to investigate if removing the different portion of the posterior part of the spine could lead to concentration of strain and stress in the intervertebral discs between the treated levels and in the adjacent intervertebral discs, potentially damaging the discs. For these reasons, one level cranial and one caudal the treated levels were included. To ensure that each disc under investigation was loaded and constrained in a relevant condition, the test specimen add to include an additional vertebra cranial and one caudal to these levels. These choices resulted in L2-Sacrum specimens.

### 4.1 About the errors and repeatability analysis

Considering the range of strains expected in the bone tissue and in the intervertebral discs, both the systematic and random errors were acceptable (Palanca et al., 2016) and in line with similar previous DIC analysis (Palanca et al., 2018; Palanca et al., 2020). The coefficient of variation between repetitions was at worst 11.3% in one configuration, and smaller in all the other configurations for all specimens, confirming that the entire test method had a good repeatability.

### 4.2 About the range of motion after hemilaminectomy and laminectomy

The experimental measurements showed that hemilaminectomy did not significantly increase the range of motion of the lumbar spine under any loading configurations (flexion, extension, lateral bending). Similar results were found by Fiss et al., 2021, in the cervical spine (Fiss et al., 2021). Conversely, the present findings showed that the removal of all the posterior arch, the flavum, and supraspinous ligaments during laminectomy caused an increase in the lumbar range of motion in flexion; in the other loading configurations, the preservation of the facet joints seemed more significant than the removal of the posterior structures for the range of motion. In common with this results, Costa et al., 2018, reported that bone-preserving laminectomy had limited detrimental biomechanical consequences (Costa et al., 2018).

Lener et al., 2023, analyzed the effect of different decompression procedures starting from the intact condition, to a fully laminectomy with a posterior fixation in a cadaver experiment (Lener et al., 2023). Similar to the present work, they found that ROM after hemilaminectomy did not significantly differ from the intact condition neither in flexion/extension nor in lateral bending, while they found a significant increase for both the flexion/extension and lateral bending after the laminectomy. Lener et al. 2023, applied a higher bending moment (7.5 Nm) than in the present study, on shorter segments (L3-S1 or L2-L5) than in the present study. Furthermore, they analyzed the entire flexion-extension cycle (rather than flexion or extension separately), and the entire right-left lateral bending (thus not discriminating ipsilateral and contralateral bending with respect to side of the hemilaminectomy). These differences between Lener et al. 2023, and the present study did not allow a direct comparison of the values of the range of motion.

Delank et al., 2010, assessed the ROM of a lumbar spine *ex vivo* after different decompressive surgical procedures under a relatively low moment (3.5 Nm, similar to the present study). To measure the range of motion, they used an ultrasound tracking system. Similar to the present findings, they found that hemilaminectomy did not significantly change the mobility of the lumbar spine in the same loading configurations tested in the present study. They also reported that one-level laminectomy did not impact the ROM, while in the present study the ROM after two-level laminectomy significantly increased in flexion. Differently from the present work, in which the ROM of the entire lumbar segment was measured, Delank et al. 2010, did not measure the range of motion of all the lumbar segment, but ROM of each single functional spinal unit. Unlike our study, they also preserved the interspinous and supraspinous ligaments in the decompressive procedures: in flexion preservation of these ligaments could be fundamental to avoid changes in the range of motion if the lumbar spine, while it did not seem to impact the other loading configurations (Delank et al., 2010).

Smith et al., 2014, found that lumbar decompression with a minimally invasive approach resulted in a significantly smaller increase in the range of motion, compared to the traditional laminectomy in case of flexion, extension and ipsilateral bending. Unlike the present study, they removed at least 15% of the facets, in both the decompressive procedures simulation (Smith et al., 2014). This could confirm the important implications of facet joint preservation on the spine stability after decompressive surgery. Conversely, Delank et al., 2010, did not find significant changes in the range of motion of lumbar functional spinal units after a bilateral facet resection (Delank et al., 2010).

The fact that the present findings on the impact of the decompressive procedures on the range of motion were in agreement with previous studies confirms the relevance of these measurements. In addition, the present study focused also on changes in the strain distribution in the intervertebral disc directly involved in the decompression, and the adjacent ones.

### 4.3 About the strains in the intervertebral discs after hemilaminectomy

In flexion, the portion of disc affected by the highest tensile (ε1) strain tended to enlarge with respect to the intact condition. In fact, during flexion, the intervertebral disc tends to bulge: at mid-height, the anulus fibrosus is stretched and the tensile strain are highest. On the contrary, the portion of disc close to the endplates, and especially to the lowest one, which were compressed, showed highest compressive (ε2) strain in flexion. No significant differences on the largest strain were found after hemilaminectomy, confirming that in flexion the removal of one lamina, preserving the facet joints did not change the strain distribution on all the intervertebral discs.

In extension, removing part or the entire posterior arch could result in an asymmetrical motion, affecting the distribution of both tensile and compressive strains on the L4-L5 intervertebral disc. The presence of the facet joints constrained the intervertebral motions: indeed, no significant differences on the largest strain were found, similar to flexion. Therefore, preserving the facet joints could help reduce the risk of damaging the intervertebral disc.

Similar strain distributions were observed on ipsilateral bending and contralateral bending before and after hemilaminectomy. The highest tensile strains (ε1) were located where the intervertebral discs stretched, and in particular at mid-height in the same side of the bending, because of the disc was bulging, and in the opposite side of the bending. The highest compressive strain (ε2) corresponded to the side where the specimens were subject to compression, on the same side of the bending. Removal of one lamina (hemilaminectomy) did not create a different strain distribution when the spine was laterally bent towards either side. This suggests that preservation of the spinous process, supraspinous and interspinous ligaments, and facet joints grants the same load transfer mechanism along the lumbar spine. Preserving these structures allowed not to generate significantly larger tensile or compressive strain, which could increase the risk of disc damage after hemilaminectomy.

Both the tensile and compressive strain distribution on the L3-L4 (cranial to the operated levels) and L5-S1 intervertebral disc (caudal to the operated levels) did not show remarkable differences, both as qualitative a pattern and in absolute terms, after hemilaminectomy with respect to the intact condition in all the loading configurations. This suggests that hemilaminectomy did not impact both the cranial and caudal levels.

### 4.4 About the strains in the intervertebral discs after laminectomy

In flexion, following the trend already reported for hemilaminectomy, the highest tensile strains (ε1) were observed in a larger portion of the disc, with respect to the two previous conditions. Similar to hemilaminectomy, the highest compressive (ε2) strains were visible in the portion of the disc close to the lowest endplate. No significant differences on the largest strains were found, confirming that in flexion the anterior and posterior ligament and the facet joint safeguarded the intervertebral discs from the risk of damaging.

In extension, no significant differences were observed on the largest strain, nor on the strain distribution, suggesting that keeping the facet joints intact preserved the kinematics of the lumbar spine.

The strain distributions for ipsilateral bending and contralateral bending after laminectomy were similar to the other two conditions (intact and hemilaminectomy). Again, the highest tensile strains (ε1) were observed at mid-height of the disc, in the same side of bending, where the intervertebral discs stretched because of bulging, and in the opposite side of the bending. The highest compressive strain (ε2) corresponded to the side where the specimens were subject to compression, on the same side of the bending.

On the L4-L5 intervertebral disc, after laminectomy, higher compressive strain (ε2) could be observed especially in lateral bending. Removal of the posterior arch possibly transferred more load on the facet joint, which came closer to each other and leading to larger compression of the L4-L5 intervertebral disc during lateral bending. This resulted also in a significant increase of the largest tensile and compressive strain.

Both Shah et al., 1978; Hongo et al., 1999, found the largest strains near the pedicles and on the *pars interarticularis* in pure compression on intact lumbar vertebrae (Shah et al., 1978; Hongo et al., 1999). Fu et al. (2017), did not find significant differences on the inferior articular process at the caudal level where the bilateral facetectomy was performed neither in flexion nor in lateral bending (Fu et al., 2017). Comparisons with the present study are difficult as Fu et al. 2017, only measured the strains at selected locations where strain gauges were applied. Moreover, in the present study the facet joints were preserved while the laminae and posterior processes where removing, thus possibly resulting in a different load transfer mechanism.

No relevant differences could be observed in the area affected by highest compressive strain (ε2) after laminectomy, compared to the he two previous conditions, on the L3-L4 intervertebral disc. Despite that, a significant increase in the largest compressive strain in ipsilateral bending was found.

Laminectomy did not significantly increase the strain on the anulus fibrosus of the L5-S1 intervertebral disc (caudal to the operated levels). This was visible also in the strain distribution maps in all the loading configurations, suggesting that laminectomy, like hemilaminectomy, would not significantly increase the risk of damaging the caudal levels.

It must be noted that, under no conditions and in no loading configurations, the strains on the intervertebral discs exceeded a value of 0.1, which is associated with physiological loads (Cristofolini, 2015). This confirms that neither hemilaminectomy nor laminectomy can be expected to increase the risk of damage of the intervertebral discs.

### 4.5 Limitations

One limitation of this *ex vivo* study is that is not possible to reliably consider the effect of muscular forces and the sagittal balance on the spine. However, the aim of this study was to compare sequentially different decompressive procedures, and each specimen was compared to itself, in the intact state, to investigate the variations. So, the biomechanical effects caused by hemilaminectomy and laminectomy could be comparatively assessed.

This study focused on flexion, extension and lateral bending as these are the most common loading configurations in the literature, and also the most critical ones in terms of risks of spinal instability as they can more easily lead to spondylolisthesis. Torsion could be another relevant loading configuration to be included. However, this scenario is less common in the literature, and was not part of our experimental protocol either. Conversely, axial compression is not relevant in assessing the stability of the lumbar spine after the decompressive treatments investigated in this study. Indeed, during axial compression, the force is mainly transferred along the spine through the intervertebral discs in the anterior part and the facet joints in the posterior part. Hemilaminectomy and laminectomy simulated in this study consisted of removal of different portion of the laminae without damaging or affecting the facet joints. Therefore, as none of the parts involved in the load distribution during the axial compression were removed, a pure compression test would probably not have shown differences between the different spine conditions. Indeed, pure compression is very seldom simulated in the literature in this kind of investigations.

Another limitation relates to the relatively low moments applied. Low moments were intentionally chosen to reduce the risk of damaging the specimen during the different repetition of each test in each loading configuration and in each condition. The magnitude of the moment applied during the present tests was towards the low end of the range reported in the literature. Despite this limitation, the effects on the range of motion found in the previous studies were comparable to those reported in the literature for similar and larger moments. It is however possible that higher moments could have a different impact on the strain distribution on the intervertebral discs.

In total 600 cycles were applied to each specimen (this count includes also all the preconditioning cycles, the different loading configurations and the different spine conditions). This was necessary, as each specimen had to be tested in all conditions to allow repeated-measures analysis. To avoid tissue damage, a relatively low peak load was applied (2.5 Nm, which is at the bottom of the range that can be found in the literature, as mentioned above). To preserve the specimens, they were kept fully hydrated throughout the test sessions. Furthermore, to ensure that no conditioning effect could affect subsequent steps of testing, the specimens were allowed to rest between repetitions for a time that was at least one order of magnitude longer than the duration of load application.

The present study focused on intervertebral disc strains, which are *one of* the predictors of risk damage (Iatridis et al., 2005; Adams and Roughley, 2006; Zielinska et al., 2021). It must be acknowledged that other factors such as cycling loading, phenotype, low hydration, aging, are contributing factors. For this reason, it is not possible to indicate a specific value of strain as a threshold for risk of disc damage. However, the distribution of strain in the disc is an important parameter which needs to be considered, together with the other factors.

Unfortunately, some analyses have been performed without the inclusion of all specimens, due to a data loss. However, in the worst case, at least six specimens have been included. Wilke et al. 1998 suggest to include at least six specimens to grant a reasonably conclusive statistical analysis. Similarly, other studies in the literature rely on six specimens (Quint et al., 1998; Lee et al., 2010; Ma et al., 2014; Smith et al., 2014; Costa et al., 2018). Therefore, we can state the statistical power of our analysis and the validity of our results.

## 5 Conclusion

Laminectomy is widely regarded as the “gold standard” surgical treatment for low back spinal stenosis, aimed at alleviating symptoms such as pain, numbness, and weakness in the lower limbs. Although less invasive procedures are theoretically advantageous in reducing iatrogenic instability and postoperative back pain, definitive conclusions are hampered by methodological flaws and inadequate reporting in existing studies. There is a lack of research on the incidence of iatrogenic instability using standardized definitions for radiological and clinical instability over comparable follow-up periods, as well as long-term outcomes (Overdevest et al., 2015).

This *ex vivo* study quantitatively assessed how different surgical decompressive techniques affect the range of motion and strain distribution in the intervertebral discs of the lumbar spine. Our findings indicate that hemilaminectomy does not significantly change the lumbar spine’s range of motion or notably increase the strain on intervertebral discs under any loading configurations. In contrast, laminectomy considerably increases the range of motion in flexion and the strain magnitude in the L4-L5 disc during lateral bending.

Thus, while rigorous clinical studies are necessary to compare decompression techniques for lumbar stenosis to formulate high-quality, evidence-based recommendations, our data provide valuable insights for surgeons to enhance surgical decision-making and procedure planning for treating lumbar spinal stenosis.

## Data Availability

The raw data supporting the conclusions of this article will be made available by the authors, without undue reservation.
