# Supplementary material for: Effect of two-level decompressive procedures on the biomechanics of the lumbo-sacral spine: an ex vivo study
Source: Front Bioeng Biotechnol. 2024 Jul 9;12:1400508. doi: 10.3389/fbioe.2024.1400508 (PMC11263119; doi:10.3389/fbioe.2024.1400508)
Supplement: Supplementary file 1 [file Table1.docx]

Supplementary Material

Effect of two-level decompressive procedures on the biomechanics of the lumbo-sacral spine:
an *ex vivo* study

**Sara Montanari, MEng, Elena Serchi, MD, Alfredo Conti, MD, PhD,**

Giovanni Barbanti Bròdano, MD, Rita Stagni, PhD, Luca Cristofolini, PhD*

*** Corresponding Author**: Luca Cristofolini, [luca.cristofolini@unibo.it](mailto:luca.cristofolini@unibo.it)

# Additional statistical analysis

Before investigating if differences among the three conditions were significant, the Shapiro-Wilk test was performed in order to assess the normality distribution of each parameter. Results of the Shapiro-Wilk test are listed in table S1.1.

**Table S1.1** –The normality of the distribution of each parameter was tested with the Shapiro-Wilk test. The distribution was normal when p>0.05.

|  |  | **Intact** | **Hemilaminectomy** | **Laminectomy** |
| --- | --- | --- | --- | --- |
| **ROM** | Flexion | 0.109 | 0.312 | 0.550 |
|  | Extension | 0.024 | 0.548 | 0.046 |
|  | Ipsilateral bending | 0.352 | 0.111 | 0.109 |
|  | Contralateral bending | 0.064 | 0.205 | 0.239 |
| **L3-L4 ε1** | Flexion | 0.164 | 0.204 | 0.481 |
|  | Extension | 0.539 | 0.239 | 0.622 |
|  | Ipsilateral bending | 0.572 | 0.068 | 0.199 |
|  | Contralateral bending | 0.641 | 0.543 | 0.440 |
| **L3-L4 ε2** | Flexion | 0.370 | 0.816 | 0.528 |
|  | Extension | 0.091 | 0.268 | 0.122 |
|  | Ipsilateral bending | 0.328 | 0.036 | 0.184 |
|  | Contralateral bending | 0.375 | 0.928 | 0.323 |
| **L4-L5 ε1** | Flexion | 0.524 | 0.070 | 0.003 |
|  | Extension | 0.001 | 0.040 | 0.001 |
|  | Ipsilateral bending | 0.101 | 0.227 | 0.188 |
|  | Contralateral bending | 0.649 | 0.494 | 0.339 |
| **L4-L5 ε2** | Flexion | 0.445 | 0.341 | 0.120 |
|  | Extension | 0.002 | 0.055 | 0.007 |
|  | Ipsilateral bending | 0.006 | 0.046 | 0.016 |
|  | Contralateral bending | 0.027 | 0.105 | 0.227 |
| **L5-S1 ε1** | Flexion | 0.835 | 0.879 | 0.921 |
|  | Extension | 0.593 | 0.553 | 0.643 |
|  | Ipsilateral bending | 0.153 | 0.353 | 0.339 |
|  | Contralateral bending | 0.199 | 0.981 | 0.550 |
| **L5-S1 ε2** | Flexion | 0.876 | 0.121 | 0.269 |
|  | Extension | 0.604 | 0.491 | 0.934 |
|  | Ipsilateral bending | 0.974 | 0.855 | 0.603 |
|  | Contralateral bending | 0.483 | 0.120 | 0.026 |
